# Application of the ACOSOG Z0011 criteria to Chinese patients with breast cancer: a prospective study

**DOI:** 10.1186/s12957-021-02242-1

**Published:** 2021-04-20

**Authors:** Yuan Peng, Miao Liu, Xianan Li, Fuzhong Tong, Yingming Cao, Peng Liu, Bo Zhou, Hongjun Liu, Lin Cheng, Jiajia Guo, Fei Xie, Houpu Yang, Siyuan Wang, Chaobin Wang, Yalin Chen, Shu Wang

**Affiliations:** 1 Breast Center, Peking University People’s Hospital, Beijing, China; 2 Radiotherapy Department, Peking University People’s Hospital, Beijing, China

**Keywords:** Breast cancer, Sentinel lymph node, ACOSOG Z0011, Chinese

## Abstract

**Background:**

Although the ACOSOG Z0011 study showed that axillary lymph node dissection (ALND) could be avoided in a specific population of sentinel lymph node-positive patients, it is not widely accepted by Chinese surgeons. We conducted a prospective single-arm study to confirm whether or not the results of Z0011 are applicable to Chinese patients.

**Methods:**

Patients conforming to the Z0011 criteria were prospectively enrolled at the Peking University People’s Hospital Breast Center from November 2014 to June 2019. The clinicopathological features of the study group were compared with those of the Z0011 study group. Lymphedema after surgery, the incidence of local-regional recurrence, and survival were analyzed.

**Results:**

One hundred forty-two patients who met the Z0011 eligibility criteria were enrolled in this study; 115 underwent sentinel lymph node biopsy (SLNB) alone. Compared with the Z0011 trial, younger patients were included (median age, 52 [26–82] years vs 54 [25–90] years; *P* = 0.03). For clinical T stage, tumor histology, hormone status, lymphovascular invasion, and the number of positive sentinel lymph nodes (SLNs), no statistically significant differences were observed. More patients received adjuvant chemotherapy and endocrine therapy in this study (90.85% vs 58.0% and 80.99% vs 46.6% respectively, *P* <0.001). A similar percentage of patients received radiotherapy, but more nodal radiotherapy procedures were carried out in our study (54.5% vs 16.9%). After a median follow-up of 29 months, only 1 patient (0.9%) had ipsilateral breast tumor recurrence, and no regional recurrence occurred.

**Conclusion:**

Our study showed that it is achievable to avoid ALND in patients eligible for Z0011 in China.

**Trial registration:**

ClinicalTrials.gov. Registration number NCT03606616. Retrospectively registered on 31 July 2018.

## Background

The American College of Surgeons Oncology Group (ACOSOG) Z0011 trial is the largest prospective, randomized controlled study comparing local control rates and overall survival rates of axillary lymph node dissection (ALND) and sentinel lymph node biopsy (SLNB) groups in patients with positive SLNs. The results of the trial showed that the 10-year local-regional control and overall survival rates for patients receiving SLNB alone were not inferior to those for patients receiving ALND [1, 2]. The results of this study had a major effect on the clinical practice of breast surgery; since 2012, the National Comprehensive Cancer Network guidelines have been continually changed to this day [3].

After the Z0011 trial, America, Australia, Europe, Japan, and other regions have verified the results in their own populations [4–10]. Although several studies using different databases revealed that ALND could be omitted based on the Z0011 strategy [11, 12], the attitudes of surgeons are controversial. According to a survey in 2018 in America, 49% of surgeons recommended ALND for 1 SLN metastasis, and 63% recommended ALND for 2 SLN metastases [13]. In China, the attitude is more negative, and only 16.6% of hospitals accepted the conclusions of the Z0011 study [14].

In 2014, we performed a retrospective analysis and found that the clinicopathological factors were not significantly different between the eligible group in China and the Z0011 cohort. These findings laid the foundation for omitting ALND in Chinese patients according to the Z0011 criteria [15]. However, no prospective clinical trial results have been reported in the Chinese population. The current study is the first to assess whether the Z0011 criteria to avoid ALND after positive SLN findings are applicable to Chinese patients with breast cancer.

## Methods

Beginning in November 2014, we adapted the results of the Z0011 trial to the management of patients with breast cancer at Peking University People’s Hospital (PKUPH) Breast Center. This was a prospective, single-arm study. To achieve 80% power for detecting a non-inferior result with a two-sided type I error rate of 0.05 according to Z0011 trial, 344 patients need to be assigned. From November 2014 to June 2019, patients with invasive breast cancer were enrolled if they met the following Z0011 trial criteria: (1) diagnosed with clinical stage T1-2N0 cancer and (2) previously underwent breast-conserving surgery with planned whole-breast irradiation. An ethics approval was issued by the Institutional Ethics Committee of Peking University People’s Hospital on 4 September 2018. The trial was registered on 31 July 2018, retrospectively registered as NCT03606616 at the following site: https://clinicaltrials.gov.

All patients received routine preoperative axillary nodal ultrasound imaging. Fine-needle biopsy was performed for suspicious lymph nodes. If the aspiration cytology suggested malignancy, ALND was performed.

SLNs were detected using blue dye and indocyanine green. All blue staining or fluorescently labeled lymph nodes were removed. Any patients with negative SLNs or isolated tumor cells within the SLNs were excluded from further analysis. ALND was performed if 3 or more positive nodes were detected or if nodes showed gross extracapsular extension. Adjuvant treatment for each patient was based on national guidelines and physicians’ choices. Whole-breast radiation therapy was performed in addition to other radiotherapy fields depending on the treatment specified by the radiation oncologists.

We collected the clinical and pathological data, including the adjuvant therapies.

Local-regional recurrence, distant metastasis, and survival were closely monitored. The presence of lymphedema was reported in one of 2 ways: (1) self-report by the patient or (2) physician diagnosis using the Breast Cancer and Lymphedema Symptom Experience Index (BCLE-SEI). BCLE-SEI has two parts. Part I assessed 24 symptoms associated with breast cancer-related lymphedema and can be used alone for early monitoring or diagnosis of lymphedema. Symptoms include impaired limb mobility, arm swelling, breast swelling, chest wall swelling, heaviness, firmness, tightness, stiffness, numbness, tenderness, pain, aching, soreness, stiffness, redness, blistering, burning, stabbing, and tingling (pins and needles). Three symptoms can be used to judge healthy women and at-risk survivors. Nine symptoms discriminate at-risk survivors with lymphedema [16].

All data were analyzed using SPSS, version 20.0 statistical software. The clinicopathological features of the study group and the Z0011 trial SLNB cohort were compared. The characteristics of the eligible group and the Z0011 SLNB alone group were compared using the chi-square test and *t* tests. A *P* value < 0.05 was considered significant. The survival data were analyzed with descriptive statistics.

## Results

**Fig. 1:**
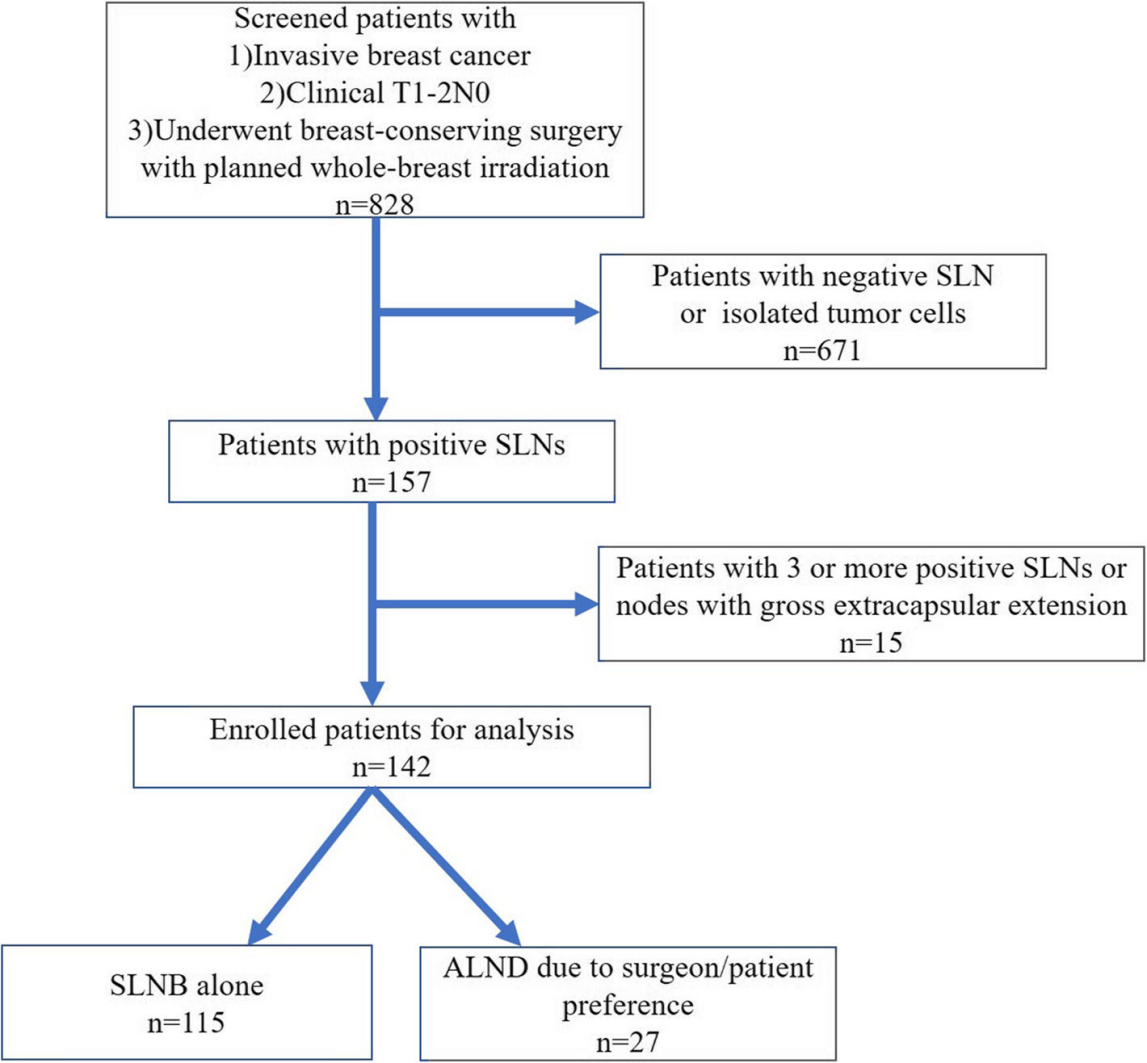
Flow chart of the study procedures

In the current study, 828 patients with invasive breast cancer were screened from November 2014 to June 2019. The patient flow chart is shown in Fig. 1. Six hundred eighty-six patients with (1) negative SLNs, (2) isolated tumor cells, (3) 3 or more positive SLNs, or (4) nodes with gross extracapsular extension were excluded from the study and underwent ALND. One hundred forty-two patients were eligible and therefore remained in the analysis. Twenty-seven patients otherwise eligible for SLNB alone underwent ALND as a result of either surgeon or patient preference.

Table 1 shows the baseline characteristics of the 142 eligible patients. The median age of the patients was 52 (range, 26–82) years old, and 112 (78.87%) patients presented with clinical T1 tumors. Patients who were hormone receptor positive accounted for 82.39%. In 76.76% of patients, only 1 metastatic lymph node was found among SLNs. Adjuvant chemotherapy was given to 129 (90.85%) patients, and adjuvant endocrine therapy was given to 115 (80.99%) patients. Of the 27 patients who underwent ALND, 7 (25.93%) had additional positive nodes (range, 1–9). Among 115 patients treated with SLNB alone, 101 (87.8%) received radiotherapy. Detailed radiotherapy records were obtained for 99 patients. All patients received three-dimensional intensity-modulated radiation therapy. Of these, 22 (22.22%) patients received whole-breast radiotherapy using standard tangential fields, 50Gy in 25 fractions, followed by a boost on the tumor bed of 10Gy in five fractions. Twenty-three (23.23%) patients received level I/II axillary radiotherapy (50Gy in 25 fractions), and 54 (54.55%) patients received nodal radiotherapy (nodal radiotherapy included the level III axillary and supraclavicular nodes, also 50Gy in 25 fractions). The features of each group of patients are shown in Table 2.

**Table 1:** Baseline characteristics of the eligible patients (*n* = 142)

Clinicopathological characteristics	Total (*n*=142)	SLNB alone (*n*=115)	ALND (*n*=27)
Age (median, range)	52 years (26–82)	52 years (29–82)	44 years (26–69)
Age group, no. (%)			
≤ 50	68 (47.89)	51 (44.35)	17 (62.96)
> 50	74 (52.11)	64 (55.65)	10 (37.04)
Clinical T stage, no. (%)			
cT1	112 (78.87)	90 (78.26)	22 (81.48)
cT2	30 (21.13)	25 (21.74)	5 (18.52)
Pathological T stage, no. (%)			
pT1	103 (72.54)	81 (70.43)	22 (81.48)
pT2	39 (33.91)	34 (29.57)	5 (18.52)
Tumor histology, no. (%)			
Ductal	119 (83.80)	95 (82.61)	24 (88.89)
Lobular	15 (10.56)	13 (11.30)	2 (7.41)
Other	8 (5.63)	7 (6.09)	1 (3.7)
Lymphovascular invasion, no. (%)			
Present	33 (23.24)	26 (22.61)	7 (25.93)
Absent	68 (47.89)	60 (52.17)	8 (29.63)
Missing	41 (28.87)	29 (25.22)	12 (44.44)
Hormone status, no. (%)			
Positive	117 (82.39)	92 (80.0)	25 (92.59)
Negative	25 (17.61)	23 (20.0)	2 (7.41)
HER2 status, no. (%)			
Negative	118 (83.10)	96 (83.48)	22 (81.48)
Positive	21 (14.79)	17 (14.78)	4 (14.81)
Unknown	3 (2.11)	2 (1.74)	1 (3.70)
Number of positive SLN, no. (%)			
1	109 (76.76)	94 (81.74)	15 (55.56)
2	33 (23.24)	21 (18.26)	12 (44.44)
Adjuvant chemotherapy, no. (%)			
Yes	129 (90.45)	102 (88.70)	27 (100.00)
No	13 (9.15)	13 (11.30)	0 (0.00)
Adjuvant endocrine therapy, no. (%)			
Yes	115 (80.99)	92 (80.00)	23 (85.19)
No	27 (19.01)	23 (20.00)	4 (14.81)
Radiotherapy, no. (%)			
Yes	127 (89.44)	101 (87.83)	26 (96.30)
No	15 (10.56)	14 (12.17)	1 (3.70)

**Table 2:** Comparison of the characteristics of patients treated with whole-breast irradiation alone, level I/II radiotherapy, and treated with nodal radiotherapy

Characteristics	Whole-breast alone (*n*=22)	Level I/II (*n*=23)	Nodal radiotherapy (*n*=54)
Age, (median) years	52.5	55	49.5
Pathological T stage, (cm, mean)	1.51	1.78	1.89
Hormone status, no. (%)			
Positive	20 (90.91)	20 (86.96)	43 (79.63)
Negative	2 (9.09)	3 (13.04)	11 (20.37)
HER2 status, no. (%)			
Negative	20 (95.24)	21 (91.30)	42 (79.25)
Positive	1 (4.76)	2 (8.70)	11 (20.75)
Unknown	1	0	1
Lymphovascular invasion, no. (%)			
Present	4 (22.22)	7 (43.75)	14 (34.15)
Absent	14 (77.78)	9 (56.25)	27 (65.85)
Missing	4	7	13
Number of positive SLNs			
One	20 (90.91)	20 (86.96)	41 (75.93)
Two	2 (9.09)	3 (13.04)	13 (24.07)

The pathological and clinical characteristics of patients in the Z0011 trial SLNB alone arm were compared to those of the patients in the current study; these comparisons are shown in Table 3. For clinical T stage, tumor histology, hormone status, lymphovascular invasion, and the number of positive SLNs, no statistically significant differences were observed. Our eligible patients were younger than those in the Z0011 trial, and most patients received chemotherapy and endocrine therapy (*P* <0.001).

**Table 3:** Clinicopathological characteristics of the patients in the Z0011 SLNB-alone arm and those in the current study

Characteristics	Eligible patients (*n*=142)	Z0011 SLNB alone (*n* = 436)	*P* value
Age, median (range), years	52 (26–82)	54 (25–90)	-
Age group, no. (%)			
≤ 50	68 (47.89)	160 (37.6)	0.03
> 50	74 (52.11)	266 (62.4)	
Missing	0	10	
Clinical T stage, no. (%)			
cT1	112 (78.87)	303 (70.6)	0.056
cT2	30 (21.13)	126 (29.4)	
Missing	0	7	
Tumor histology, no. (%)			
Ductal	119 (83.80)	356 (84.0)	0.589
Lobular	15 (10.56)	36 (8.5)	
Other	8 (5.63)	32 (7.5)	
Missing	0	12	
Hormone status, no. (%)			
Positive	117 (82.39)	328 (83.7)	0.726
Negative	25 (17.61)	64 (16.3)	
Missing	0	44	
Lymphovascular invasion, no. (%)			
Present	33 (32.67)	113 (35.2)	0.641
Absent	68 (67.33)	208 (64.8)	
Missing	41	115	
Number of positive SLNs, no. (%)			
0–1	109 (76.76)	324 (78.1)	0.746
≥ 2	33 (23.24)	91 (21.9)	
Missing	0	21	
Adjuvant chemotherapy, no. (%)			
Yes	129 (90.85)	253 (58.0)	< 0.001
No/missing	13 (9.15)	183 (42.0)	
Adjuvant endocrine therapy, no. (%)			
Yes	115 (80.99)	203 (46.6)	< 0.001
No/missing	27 (19.01)	233 (53.4)	
Radiotherapy, no. (%)			
Yes	127 (89.44)	277* (89.64)	0.947
No	15 (10.56)	32* (10.36)	

*n=309

No axillary recurrences have occurred in our study at a median follow-up of 29 months (range, 5–60 months). One patient had ipsilateral breast tumor recurrence 56 months after the operation.

We administered a questionnaire using the BCLE-SEI to all patients. Thirteen patients were at risk of lymphedema (3–8 symptoms). The scores in the ALND group were higher than those in the SLN-alone group (Table 4). Of the patients in the SLN-alone group, 2 (1.7%) were either self-reported cases of lymphedema or had a physician diagnosis of lymphedema. These two patients received nodal radiotherapy (level III axillary and supraclavicular nodes). Three (11.1%) patients who had ALND reported lymphedema. All patients who self-reported or had physician diagnosis of lymphedema received nodal radiotherapy. According to the Radiation Therapy Oncology Group (RTOG) acute radiation morbidity scoring criteria, grade 1 and grade 2 radiotherapy-related skin complications were much the same between different radiotherapy groups (*P*=0.445), no grade 3 or 4 level morbidities. In the axillary level I/II radiotherapy group and nodal radiotherapy group, one patient each had a wound dehiscence without infection.

**Table 4:** The BCLE-SEI symptoms scores of the eligible patients

Number of symptoms	SLNB-alone (*n*=115)	ALND (*n*=27)	*P v*alue
0–2, *n* (%)	107 (93.04)	22 (81.48)	<0.001
3–8, *n* (%)	8 (6.96)	5 (18.52)	
≥9, *n* (%)	0	0	

## Discussion

In recent years, the clinical practice of breast surgery has been greatly influenced by the ACOSOG Z0011 trial results. In Europe and Australia, the rate of ALND obviously decreased after the Z0011 study was published [17, 18]. In China, ALND was the standard treatment for patients with positive SLNs until the guidelines of the Breast Cancer Committee affiliated with the Chinese Anti-Cancer Association were revised in 2019 [19]. The reason why the Z0011 trial results have not been accepted by most Chinese surgeons in the past few years may be because of the lack of Chinese patients’ own data. It is unknown whether Chinese patients with breast cancer and patients with breast cancer from the West share clinical characteristics similar to those of the Z0011 trial. It is also unclear whether results similar to those of the Z0011 trial, especially excellent local-regional control, can be achieved in a population of Chinese patients under the current adjuvant treatment pattern in China. In this study, we prospectively investigated whether or not the Z0011 criteria could feasibly be applied to Chinese patients.

In our prospective study, 142 patients met the ACOSOG Z0011 eligibility criteria, and 115 patients avoided undergoing ALND. Although our patients were slightly younger than the patients in the Z0011 trial, the clinical T stage, tumor histology, hormone status, lymphovascular invasion, and the number of positive SLNs showed no remarkable differences between the two studies. In our study, 25.94% (7 out of 27) of patients had additional positive nodes after ALND, similar to that in previous studies [5–7, 15, 20] (Table 5). Although a 1 out of 4 probability of non-SLN metastasis exists, we still achieved very good local-regional control and survival. Only 1 patient experienced ipsilateral breast recurrence, and no regional recurrences or deaths occurred. This result is consistent with that of the Z0011 trial, which showed that potential residual positive lymph nodes could be successfully controlled by radiotherapy and systemic therapies. Therefore, ALND can be avoided in a large majority of patients with positive SLNs following the Z0011 criteria. In our study, ALND was avoided in 73.25% (115 out of 157) of positive SLNs, similar to other retrospective and prospective reports [5, 7, 10, 20, 21].

**Table 5:** Additional positive nodes after ALND in eligible patients based on the Z0011 criteria

Author	Number of patients	Non-SLN positive
Giuliano et al. [1]	355	27.3%
Aigner et al. [6]	132	39%
Delpech et al. [5]	87	29%
Liu et al. [15]	151	25.2%
Verheuvel et al. [20]	625	26%
Ngui et al. [6]	22	27.3%
Present study	27	25.94%

There were differences between patients in the Z0011 trial and those eligible for our analysis. First, most studies used radioisotopes, blue dye, or, in the case of Japan, indocyanine green and technetium tin colloids for SLNB [5, 6, 8]. Our study used blue dye and indocyanine green. According to previous research reports, indocyanine green in conjunction with blue dye is an efficient method [22, 23] to detect SLNs without affecting the results.

Second, we conducted a rigorous preoperative assessment. The Z0011 trial applied no specific requirements for preoperative axillary lymph node imaging assessment, unless the enlarged axillary lymph nodes were palpable, according to the US guidelines. However, in our opinion, the imaging assessment of axillary lymph nodes before surgery may be useful. If a lymph node is found to be positive on ultrasound-guided fine-needle biopsy, a higher nodal burden is predicted than for a positive SLNB [20, 24, 25]. Under the European guidelines [26, 27], in patients with or without palpable lymph nodes, axillary ultrasound is a routine diagnostic procedure. This is true according to the Chinese guidelines as well [19]. According to the guidelines in China, our study required the preoperative assessment of the axillary nodal status of all patients using ultrasound. If fine-needle aspiration cytology for suspicious lymph nodes was positive, then, the patients were ineligible for enrollment. However, in Morrow’s study, the researchers excluded routine imaging of the axilla with ultrasound or magnetic resonance imaging [4]. In their opinion, even if the image-guided aspiration was positive, there were still some patients with only 1 or 2 positive nodes who were able to avoid ALND; further, the local-regional control was unaffected. Moreover, in our previous study, we assumed that if only one abnormal lymph node is detected on ultrasound, then, fine-needle biopsy could be omitted, but not for multiple suspicious nodes [28]. Perhaps axillary ultrasound assessment and fine-needle aspiration are not necessary for all patients, but we currently cannot omit the use of either in China.

**Fig. 2:**
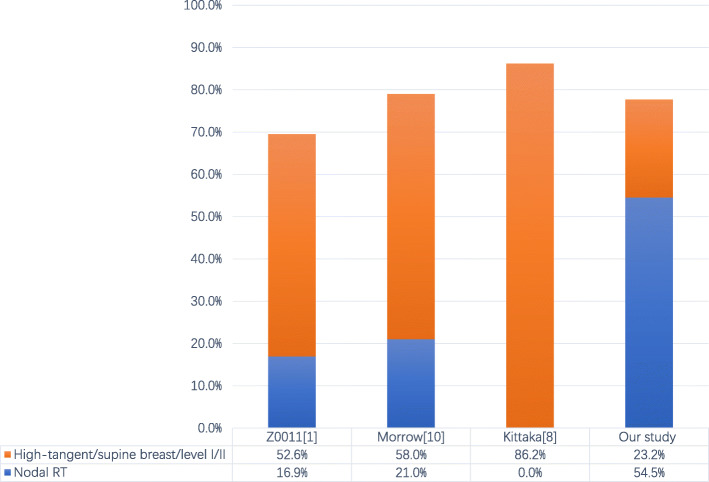
Radiotherapy field in the prospective studies

Third, in practice, more patients received regional nodal radiotherapy. In the Z0011 trial, among the patients with radiotherapy records, 52.6% were treated by high tangent radiotherapy, and 16.9% received treatment in the supraclavicular region [29]. In Morrow’s study, 21% of the patients received breast and nodal irradiation, and 58% received supine breast radiotherapy (this method allows patients to receive more axillary I/II radiotherapy than prone breast radiation therapy). Meanwhile, 23.2% of our patients received level I/II axillary irradiation, and 54.5% were treated with nodal radiotherapy (level III axillary and supraclavicular nodes). The radiotherapy field in several prospective studies is shown in Fig. 2. The choice of irradiation field is directly related to the understanding of recurrence risks by radiation physicians. Patients with high risks of recurrence, including those with a young age, larger tumor size, hormone receptor negative status, and HER2 positive status, were more likely to receive nodal RT in our study. The use of nodal RT increased with the number of positive SLNs; this is consistent with the Z0011 trial and Morrow’s study. High-risk patients treated with heavier radiation have also been confirmed in a nomogram-based study [30]. We hypothesized that radiation oncologists, who could not be blinded to the surgical treatment of the patients or to the pathological results after surgery, may have treated patients in the SLN-only arm with high-tangent radiotherapy to include a component of axillary level I/II and even three-field radiation more often than those in the ALND arm, particularly for patients at high risk. Even in America, nearly half of Z0011-eligible patients receive regional nodal irradiation according to the National Cancer Database study [31]. Close multidisciplinary teamwork between clinical oncologists and radiation physicians is needed to optimize the radiotherapy field. The optimal radiation therapy for these patients still needs further study.

Fourth, more patients in our study received chemotherapy and endocrine therapy than those in the Z0011 trial (*P* < 0.001). Chemotherapy and endocrine therapy can improve the prognosis of patients with breast cancer, whether in terms of locoregional control or overall survival. Positive lymph nodes are one of the indications for adjuvant chemotherapy per the Chinese Society of Clinical Oncology guidelines. The use of prognostic multigene signatures such as Oncotype DX or MammaPrint may influence results in the future in China. We believe that reducing chemotherapy in some low-risk patients will not affect the prognosis of these patients. The proportion of endocrine therapy in our study is almost the same with previous study other than Z0011 trial. And all hormone receptor-positive patients receiving endocrine therapy is the standard therapy in all guidelines.

The use of SLNB alone resulted in fewer complications. In the Z0011 trial, lymphedema was reported by 13% of patients after ALND and by 2% of patients after SLNB alone at 1 year. The incidence of lymphedema diagnosed by arm circumferences (defined as a 2 cm or greater postoperative increase in ipsilateral arm measurements compared with the contralateral arm) was 6% vs 11% in the 2 arms, respectively [32]. The lymphedema evaluation methods varied in different studies. In the IBCSG 23-01 trial, the treating physician reported edema, and assessments were based on the National Cancer Institute Common Toxicity Criteria version 2. The incidence of lymphedema was 3% in the SLN group and 13% in the ALND group (median follow-up of 5 years) [33]. Our patients received more nodal RT than those in the Z0011 trial, but we did not see a significant increase in the incidence of lymphedema. The incidence of lymphedema reported by patients or physicians was 11.1% after ALND and 1.7% after SLNB alone with a median follow-up of 29 months. In a trial comparing radiotherapy and ALND after SLNB, at 5 years, the incidence of lymphedema reported by arm circumference was 5% in the radiotherapy arm and 13% in the ALND arm [34]. From the above data, even after receiving axillary radiotherapy after SLNB, the incidence of edema did not increase significantly, and the proportion of edema was significantly reduced compared to that after ALND. However, a longer follow-up period is needed.

This study has several limitations. First, the median follow-up of 29 months was short, and the number of patients was insufficient to draw final conclusions about the incidence of local-regional recurrences. However, thus far, our results demonstrate an extremely low rate of local-regional recurrence in Chinese patients considering the diagnosis and treatment pattern today. Long-term follow-up is suggested to confirm the reliability of our data. Although the number of patients enrolled and follow-up time have not been reached as designed, but the wonderful survival data has verified the safety of the application of the Z0011 criteria to some extent. Our current results show that the survival data is much higher than expected, the 2-year OS is 100% and DFS 99%; so far, our center is still enrolling patients, and the follow-up is continuing. However, even if we had accrued the targeted 344 patients, 60 months of follow-up would not be enough to observe targeted events. Second, our results reflect a single-center experience, and the adjuvant treatments are influenced by individual physicians’ preferences, such as the use of adjuvant chemotherapy and the irradiation field. The results of this trial should be confirmed by multicenter studies.

To our knowledge, this prospective study is the first to apply the ACOSOG Z0011 criteria to Chinese patients with early-stage breast cancer. Our study demonstrates (1) a low risk of local-regional recurrence and (2) a good prognosis in patients with positive SLNs who were treated with SLNB alone. We believe that the results of our pilot study regarding the Chinese patient population will have a great effect on the clinical practice of Chinese surgeons in treating patients with breast cancer.

## Data Availability

The datasets used and/or analyzed during the current study are available from the corresponding author on reasonable request.

## Abbreviations

ALND: Axillary lymph node dissection
SLNB: Sentinel lymph node biopsy
SLN: Sentinel lymph node
PKUPH: Peking University People’s Hospital
BCLE-SEI: Breast Cancer and Lymphedema Symptom Experience Index
